# Position paper: timely interventions in severe acute pancreatitis are crucial for survival

**DOI:** 10.1186/1749-7922-9-15

**Published:** 2014-02-10

**Authors:** Panu Mentula, Ari Leppäniemi

**Affiliations:** 1 Meilahti hospital, Department of Abdominal Surgery, University of Helsinki, Haartmaninkatu 4, PO Box 340, 00029 Helsinki, HUS, Finland

## Abstract

Severe acute pancreatitis has high mortality, but multiple and timely interventions can improve survival. Early in the course of the disease aggressive fluid resuscitation is needed for the prevention and treatment of shock. In conjunction with leaking capillaries this results in increased tissue edema, which may lead to intra-abdominal hypertension and abdominal compartment syndrome. Invasive hemodynamic monitoring is essential for optimizing fluid therapy while monitoring of intra-abdominal pressure is necessary for identification patients at risk of developing abdominal compartment syndrome. Abdominal compartment syndrome develops usually within the first days after hospitalization. Conservative treatment modalities are useful in prevention but also in the treatment of abdominal compartment syndrome. If conservative management fails surgical decompression of abdomen may be needed. Multiple organ dysfunction syndrome and increased intra-abdominal pressure predispose patients with severe pancreatitis to secondary infections. Extrapancreatic infections predominate during the first week of the disease, whereas infection of pancreatic necrosis usually develops later. Early enteral nutrition reduces the risk of infections whereas advantage of prophylactic antibiotics is lacking evidence. Surgery for infected pancreatic necrosis is associated with high mortality when performed within the first two weeks of the disease. Therefore surgery should be postponed as late as possible, preferably later than four weeks after disease onset.

## Introduction

In the majority of patients acute pancreatitis is a mild self-limiting disease. About fifteen percent of the patients develop severe disease defined by development of persistent organ failure [1]. The mortality in acute pancreatitis is mainly associated with multiple organ failure [2] whereas the risk of dying is minimal in patients with no or transient organ dysfunction [3,4]. In acute pancreatitis, multiple organ failure is a consequence of excessive activation of a systemic inflammatory response cascade [5]. Inflammatory mediators induce end-organ endothelial cell activation leading to increased permeability [6]. Leaking microvessels cause a loss of intravascular fluid and in conjunction with vasodilatation lead to hypotension and shock. Accumulation of inflammatory cells in tissues, increased interstitial fluid and activation of coagulation with microvascular thrombosis further impair oxygen supply of tissues. Clinical manifestation of all this is a multiple organ dysfunction syndrome (MODS), which develops early during the course of acute pancreatitis. Over half of the patients with severe pancreatitis have signs of organ dysfunction on hospital admission [3] and most of the organ dysfunctions develop within the first four days after admission [7]. Over half of the deaths occur within the first week from onset of the disease, and deaths usually occurred within a week after manifestation of MODS [8].

Treatment modalities of MODS are supportive including fluid replacement therapy, vasopressors, mechanical ventilation and renal replacement therapy when necessary. In patients with acute pancreatitis, abdominal compartment syndrome (ACS) may aggravate MODS, and therefore, monitoring of intra-abdominal pressure (IAP) is crucial for identification of patients at risk of ACS [9]. Development of ACS should be prevented, if possible, by conservative methods. Surgical decompression is the last but the most effective way to decrease IAP and should not be postponed too late if patient has developed ACS [10].

Patients with acute pancreatitis have a considerable risk for developing secondary infections including bacteremia, pneumonia and infection of pancreatic or peripancreatic necrosis. Extrapancreatic infections occur predominantly during the first week of illness, whereas pancreatic necrosis becomes infected later [11]. The mortality is very high in patients with persistent organ failure complicated with infected pancreatic necrosis [12]. Development of bacteremia and infected pancreatic necrosis are associated with MODS. Intestinal dysfunction plays an important role and bacterial translocation from intestine is considered the main mechanism of infection. Impaired host response systems may also predispose to clinical infections. Early enteral nutrition has been shown to reduce systemic infections [13], whereas the results from randomized trials with prophylactic antibiotics have been inconclusive [14]. Surgery is considered necessary for adequate source control when pancreatic or peripancreatic infection develops. However, because surgery for pancreatic necrosis within the first 2–3 weeks from disease onset is associated with high mortality, surgery should be postponed as late as possible [15]. Sometimes percutaneous drainage of fluid from infected acute necrotic collection may be helpful and is preferable first-line treatment for infected pancreatic necrosis during the first three weeks of illness [16].

### Fluid resuscitation and abdominal compartment syndrome

Aggressive fluid therapy during the early phase of acute pancreatitis has been traditionally the cornerstone of treatment [17]. The rationale of fluid therapy is to correct hypovolemia caused by third space fluid loss. High admission hematocrit (above normal reference limits) may serve as a marker of hemoconcentration, and it is present up to 60% of patients who develop organ failure [18], but the marker is too unspecific for predictive purposes [19]. Fluid resuscitation decreases hematocrit, which could be used as resuscitation end-point. Too aggressive resuscitation may lead to inappropriate hemodilution and very low hematocrit values (<30%) may be harmful for the patients by increasing the risk of sepsis and death [20]. Moreover, excess volume loading may increase IAP and lead to development of intra-abdominal hypertension (IAH) and abdominal compartment syndrome [21]. In patients with acute pancreatitis, hematocrit and central venous pressure as resuscitation end-points are poor indicators of volume depletion [22]. Urine output (≥0.5 ml/kg/h) may serve as another resuscitation end-point, but other modalities are needed for volume management if oliguria persists after initial volume loading. According to recent systematic review there is little evidence concerning the rate of infusion, optimal resuscitation end-point and the type of fluid in patients with acute pancreatitis [23].

In those patients with pancreatitis who develop shock the same management guidelines as for septic shock patients can be applied. These include initial fluid challenge with crystalloids (rate 1000 ml/ hour) with minimum of 30 ml/kg and administration of vasopressor epinephrine to maintain adequate blood pressure [24]. Principles of early goal directed resuscitation with monitoring of CVP, MAP and either central venous oxygen saturation or mixed venous oxygen saturation [25] can be used also in acute pancreatitis. Frequently elevated IAP should be monitored and taken into account when considering resuscitation end-points [26]. Abdominal perfusion pressure (APP) could serve as a good resuscitation end-point in patients with IAH [27]. Maintaining APP above 50–60 mmHg is recommended in order to provide sufficient perfusion to abdominal organs [28]. Lactate level should be monitored and resuscitation should be targeted to normalizing the lactate level. As soon as resuscitation end-points are reached, the infusion rate should be slowed down in order to avoid fluid overloading.

Although the use of colloids can reduce overall volume needed for resuscitation, and thus, could decrease the risk of developing IAH, the use of colloids is not recommended in the guidelines of severe sepsis and septic shock [24]. Hydroxyethyl starch (HES) does not provide any benefit compared with normal saline and its use is associated with increased need for renal replacement therapy [29].

In severe pancreatitis IAH develops as a result of fluid resuscitation and capillary leakage. Fluid accumulates into retroperitoneal space, ascites may form and tissues edema develops. In addition, paralytic bowel can contain substantial amounts of fluid and air. All this takes space in the abdominal cavity, which causes distension of the abdominal wall. Abdomen can tolerate increased volume to some extent, but when abdominal wall becomes distended increasing intra-abdominal volume cause elevation in IAP. When IAH (IAP ≥12 mmHg) develops conservative methods should be applied to prevent development of ACS. These include restriction of intravenous fluids if possible, gastrointestinal decompression with nasogastric tube, and drainage of ascites fluid [30]. Abdominal wall compliance can be increased with adequate pain management; intubation and sedation usually decreases IAP and sometimes even neuromuscular blockade can be used for this purpose. An effective way to correct positive fluid balance and prevent development of ACS is early introducing of hemofiltration [31].

Renal function is impaired already at IAP level as low as 12 mmHg [32]. In patients with established IAH, IAP and APP should be monitored. A patient with shock can easily have inappropriately low APP (<50 - 60 mmHg) even with moderate IAH. Poor perfusion increases bowel mucosal injury [33], which is associated with infectious complications and organ failure in patients with pancreatitis [34]. IAH may play significant role in ischemic bowel complications [35]. Colonic necrosis [36] but also ischemic small bowel [37] can sometimes complicate to course of severe pancreatitis, but the role of IAH in these complications has not been studied. ACS probably plays a major role in early mortality caused by multiple organ failure in acute pancreatitis. Our own observation supports this: Pancreatitis patients with ACS had severe multi organ failure early during the course of the disease and early surgical decompression was associated with reduced mortality and none of the patients treated with decompression died during the first week [10].

In most cases adequate and timely conservative management including ascites drainage [30] is successful, but if ACS develops despite these interventions, surgical decompression should be done without a delay. Midline laparostomy that allows inspection of bowel viability is recommended in order to diagnose possible ischemic lesions. In acute pancreatitis surgical decompression usually leads to open abdomen of several weeks duration [10]. Vacuum assisted closure with mesh mediated fascial traction is a superior temporary abdominal closure method with low frequency of giant hernias [38,39].

### Nutrition

There are no indications for fasting in pancreatitis. Although pancreatitis patient may have nausea and vomiting early during the course, these symptoms usually resolve rapidly. In patients with mild acute pancreatitis oral feeding can be started as soon as patient tolerates food; early oral feeding has been associated with faster recovery and shorter hospital stay [40]. In pancreatitis enteral feeding is superior to parenteral feeding. Enteral nutrition prevents bacterial overgrowth in the intestine and reduces bacterial translocation [41]. In pancreatitis enteral nutrition reduces significantly systemic infections, organ dysfunction and mortality [13,42]. Critically ill patients are typically at risk of malnutrition [43] and therefore nutrition of patients with acute pancreatitis should be initiated as soon as possible. Initiation of enteral feeding seems to be critical in pancreatitis; if delayed for more than 48 hours, the benefits from enteral feeding are lost [44,45]. The route of enteral feeding can be either gastric or post pyloric. Gastric feeding succeeds in most of the patients, and therefore feeding can be initiated by using a nasogastric tube [46]. Delayed gastric emptying may cause problems, and therefore gastric residual volume should be monitored every six hours. It is recommended that tube feeding is started with low infusion rate (10 ml/h) and increased by 10 ml/h until every six hours providing that gastric residual volume is below 250 ml [43]. This should be continued until target volume of enteral nutrition is achieved. If gastric emptying is problem prokinetics may help but better option is to place nasojejunal feeding tube, which usually resolves the problem. If patient does not tolerate required volume of enteral nutrition, parenteral nutrition can be combined with enteral so that caloric needs are fulfilled.

### Infections and the use of antibiotics

A quarter of patients with acute pancreatitis develop infectious complication [11]. Patients with severe acute pancreatitis are more susceptible to develop infections [11]. Patients with organ dysfunctions have higher incidence of bacterial translocation [34]. They also have impaired immune system [7]. The majority of infections are extrapancreatic such as bacteremia and pneumonia. The half of these infections develop within the first week post admission [11]. Diagnosis of infected pancreatic necrosis is usually done significantly later, the peak incidence is between the third and fourth week from the onset of symptoms [11,47]. However, the actual contamination of necrosis happens probably much earlier [48]. Organ failure, early bacteremia and the extent of pancreatic necrosis are associated with increased risk of infected necrosis [11]. Diagnosis of infected pancreatic necrosis is challenging. Clinical signs of sepsis are too unspecific for definitive diagnosis and CT-scan shows gas bubbles in the necrotic collection in less than ten percent of patients [49]. Fine needle aspiration with bacterial culture has a substantial rate (20-25%) of false negative results, and thus, is not reliable to rule out infection [50].

Prophylactic antibiotics have been studied in many randomized trials with conflicting results and according to several meta-analyses and systematic reviews there is no evidence that patients benefit from prophylactic antibiotics [14,51,52]. However, there has been a nonsignificant trend for lower mortality and reduced number of infections, especially extrapancreatic infections in patients treated with prophylactic antibiotics. The randomized trials have been conducted with small samples sizes and some studies included a substantial number of patients with mild pancreatitis [53] with minimal risk of mortality and low risk of infectious complications. Although trials have not provided evidence that prophylactic antibiotic are effective they have not proved that they are not effective [54]. Taken together the limitations of the trials and the fact that patients with organ failure are susceptible to infections, we believe that the use of prophylactic antibiotic in patients with severe pancreatitis is justified. High incidence of infections in patients with severe pancreatitis and worse survival in patients who develop infection supports this policy. Indication for initiation of prophylactic antibiotics should be based on clinical judgment. Systemic inflammatory response syndrome (SIRS) [4], signs of organ dysfunction, presence of IAH [55], hyperglycemia, low plasma calcium or high creatinine [56] could be helpful in predicting severe disease.

If prophylactic antibiotics are not given, empiric use of antibiotics is encouraged in patients who develop organ dysfunctions, because there is high risk of bacteremia during the first week. Patients with severe pancreatitis fulfill the criteria of severe sepsis in case of infection and there is no rapid and reliable diagnostic method available to rule out infection. Delayed administration of antibiotics has been shown to worsen survival in patients with severe sepsis with or without septic shock [57]. After the end of the second week, empiric antibiotics may be needed for treatment of infected pancreatic necrosis if sepsis continues or the patient does not recover. Empiric antibiotics at this stage should cover potential pathogens including gram negative rods and gram positive cocci [47]. The role of empiric antifungals is not clear. Fine needle aspiration for microbiological samples should be taken if infected necrosis is suspected, although negative samples do not rule out infection [50]. Positive samples help in selection of antimicrobials and initiation of possible antifungal therapy. Prophylactic or empiric antibiotic should be discontinued when the patient recovers from organ dysfunctions and there is no evidence of infection.

### Surgery for infected necrosis

Infected pancreatic or peripancreatic necrosis has traditionally been considered an indisputable indication for surgical debridement [58]. Infected necrosis is a significant source of sepsis and removal of devitalized tissue is believed to be necessary for control of sepsis. However, infection usually continues after necrosectomy, especially if necrotic tissue is left in place. Before demarcation of necrosis develops, usually after 4 weeks from disease onset, it is impossible to remove all necrotic tissue without causing hemorrhage. Early surgical debridement has been associated with high risk of hemorrhage leading to increased organ dysfunction and death. If necrosectomy for infected pancreatic necrosis is done within the first two weeks the mortality rate is 75%, but gradually decreases to 5% when done later than four weeks after the onset of symptoms [15,50,59]. Multiple organ dysfunction increases mortality risk considerably in patients with infected necrosis. The mortality rate increases in proportion to the number of failed organs [50]. Infected pancreatic necrosis does not cause significant risk of death in absence of organ dysfunction [12,50]. Because high mortality is associated with early surgery and multiple organ dysfunction, it is recommended that surgery for infected necrosis should be postponed as late as possible, preferable later than four week from disease onset. Percutaneus drainage of the liquid component of the infected acute necrotic collection may serve as a bridge to surgery [16]. Sterile collections do not need drainage. Placement of a drain into a sterile necrotic collection can result in secondary infection, and a prolonged drainage may increase the risk further [60,61].

Infected pancreatic necrosis may cause worsening of multiple organ failure between the second and fourth week of the disease. Although delayed operative treatment is associated with lower mortality rate [62], it is not always possible to postpone surgery, if the condition of the patient deteriorates. Indeed, patients operated on between days 14 and 29 from admission have significantly higher prevalence of organ failure than patients operated on later than day 29 from admission [62], which may partly explain differences in mortality. There are no randomized studies comparing operative treatment and catheter drainage in this subgroup of patients with worsening multiple organ failure after two weeks from disease onset. The only randomized trial comparing open necrosectomy and minimally invasive step-up approach included only 28 (32%) patients with multiple organ failure and the median time of interventions was 30 days from disease onset [63]. In this study, the mortality rate was the same between the groups. Unfortunately, no data of subgroup analysis of patients with multiple organ failure was shown [63]. Although the use mini-invasive techniques are increasingly used for infected pancreatic necrosis, the lowest published mortality rate in patients operated on for infected necrosis is with open debridement and closed packing with 15% mortality [50]. In patients without preoperative organ failure, minimally invasive necrosectomy is associated with fewer new-onset organ failure than open surgery [63]. However, a considerable number of patients are not suitable for mini-invasive surgery either because of localization of the necrotic collection or because intra-abdominal catastrophe needs to be excluded [64].

## Recommendations

The management of patients with acute pancreatitis depends on duration of the disease. The following guidelines are provided for specific time frames.

### A. On admission

1. Diagnosis of acute pancreatitis is completed. Use CT-scan without contrast in case of diagnostic uncertainty.

2. Initiate fluid resuscitation with crystalloids for correction of hypovolemia with simultaneous monitoring of vital organ functions including IAP monitoring.

3. Assess severity based on clinical judgment and initiate prophylactic antibiotics in patients with probable severe pancreatitis.

4. If patient has any signs of organ dysfunction consider intensive care admission.

### B. Within the first 48 hours from admission

1. Re-assess the severity daily and discontinue prophylactic antibiotics in patients with mild or moderate pancreatitis.

2. Continue monitoring of vital organ functions and IAP in accordance with fluid therapy. Optimize fluid therapy. Reduce the infusion of crystalloids, if a patient is hemodynamically stable and does not show signs of dehydration.

3. If the patient has signs of deteriorating organ functions consider intensive care admission in order to start invasive hemodynamic monitoring and critical care.

4. In patients with IAH, calculate APP and use conservative efforts to prevent development of ACS.

5. In case ACS develops or the patient has low APP, use all possible conservative efforts to reduce IAP and correct APP, but if not effective perform surgical decompression with midline laparostomy.

6. Take blood cultures and plain chest radiographs for detection of infections.

7. Initiate enteral nutrition.

### C. Within the first week from disease onset

1. Same as B1 – B6.

2. Continue enteral nutrition, target for total caloric needs through enteral route.

3. Perform contrast enhanced CT-scan on days 5–7 after disease onset in patients with normal renal function. The amount and localization of necrosis may help in predicting the need of follow-up for late complications.

### D. During the second week from disease onset

1. Continue supportive care; try to get rid of excessive third space fluids if possible.

2. If the patient is septic at end of the second week or later, consider repeat CT-scan with image guided FNA and after that consider empiric antibiotics for possible infected pancreatic necrosis.

3. If infected necrosis is diagnosed, image guided percutaneous drainage of collection should be done.

4. An alternative to FNA is to put a percutaneous drain directly into the collection and take samples, however, if cultures are negative the drain should be removed as prolonged drainage may cause increased risk for infection.

5. Surgery for infected pancreatic necrosis should be avoided during the first two weeks, because necrosis is not well demarcated and surgery it is associated with high risk of hemorrhage and high mortality.

### E. After the second week from disease onset

1. Same as D1 – D4, repeat CT-scan if patient deteriorates; repeat CT-scan weekly if the patient is not recovering.

2. Percutaneus drainage of infected pancreatic necrosis can be continued if the patient shows signs of recovery, some patients may even avoid surgical treatment.

3. If the patient is deteriorating despite of setting of percutaneous drainage, proceed to surgical necrosectomy, whether there is proven infection or not.

4. In patients who do not recover but are stable, surgery for pancreatic necrosis is possible, but should be postponed as late as possible, preferably later than 4 weeks after disease onset. CT-scan before surgery is recommended for localization of necrosis and to confirm the demarcation of necrosis.

## Competing interests

The authors declare that they have no competing interests.

## Authors’ contribution

PM wrote the initial draft. AL made critical revisions, both authors read and approved the manuscript.
